# Commentary: Working with patients' treatment expectations – what we can learn from homeopathy

**DOI:** 10.3389/fpsyg.2024.1477034

**Published:** 2024-12-16

**Authors:** Simonetta Bernardini, Andrea Dei

**Affiliations:** ^1^Italian Society of Homeopathy and Integrated Medicine (SIOMI), Firenze, Italy; ^2^Department of Chemistry, Lamm Laboratory, INSTM Research Unit, University of Florence, Florence, Italy

**Keywords:** homeopathy, placebo, treatment expectation, evidence-based medicine, integrative medicine

## Introduction

In a recent work Wilhelm et al. (2024) propose a therapeutic model based on the placebo effect, as they believe occurs in the practice of homeopathic medicine, according to their literature data, which suggest that homeopathic medicines contain no molecules of active principle. We do not wish here to discuss the correctness or otherwise of the proposed therapeutic model, as it is outside our expertise. Rather, we want to highlight that the hypothesis underlying their reasoning is profoundly questionable: indeed, following their view, there is no relationship between the efficacy of the therapy and the specific character of the homeopathic remedies. It is here emphasized that this relationship exists as recent available meta-analyses have shown (e.g., Hamre et al., 2023). We want to highlight that the hypothesis of Wilhelm et al. underlying their reasoning is not supported by scientific data. A detailed analysis of this proposition can be found in the recent literature (Weiermayer et al., 2022).

## Discussion

There is no doubt that the debate on homeopathy has been conducted for decades by both proponents and their critics on the basis of prejudice and nonsense, given that experimental data were very limited. Added to this consideration, it is worth mentioning that the misinterpretation of the essence of their own therapeutic modality, as many homeopaths still today are proposing, does not stimulate industries to promote the necessary investments in appropriate research programs: the lack of a well-defined scientific platform has strongly limited the development of the discipline. However, now the situation is profoundly different, and even a superficial examination of the recent scientific literature provides clear evidence in that sense (Dei, 2020, 2024). Indeed, experimental data suggest that the therapeutic methodology should be widely reviewed in its essence, although this perspective is poorly understood and poorly received by both parties. For simplicity, we remind the authors that, contrary to what is postulated in the first words of their work, it has been shown that the ultra-diluted solutions of homeopathic remedies are found to contain a number of active principle molecules of the order of magnitude of the cells of our organism (Chikramane et al., 2012). In a recent report the authors were able to obtain the diffractometric crystal structure of the drug molecules populating a formally believed 10^−60^ M solution (Rath et al., 2024). Further, such solutions are capable of inducing relevant biological effects concerning both gene expression and the defence response of individual cells (Khuda-Bukhsh, 1997; Bigagli et al., 2014), which to our knowledge are not characterized by the placebo effect. These results have been obtained using both the DNA-array technique and appropriate chemical-physical investigation techniques, and the experimental data are indisputable. Furthermore, studies conducted at various dilutions show that the response of the biological substrate to the disturbance induced by the drug generally follows a hormetic mechanism (Dei and Bernardini, 2015), a biphasic response that, at a psychological level, of which the authors are undoubtedly experts, is difficult to justify.

These statements, now accepted by the scientific literature, in fact, nullify the validity of the starting hypothesis of the model proposed by Marcel Wilhelm and colleagues, who, despite their claims to be inspired by EBM, demonstrate that they do not know the first rule of the scientific method, which favors the exact over the claimed true in the definition of reality. Even if, and it is not up to us to say, the scientific experiment indicates that the human mind is capable of inducing the overexpression of certain genes and the underexpression of others (Seetharaman et al., 2021), a fact that is well-known to be exploited in healthcare practice, including the so-called integrated medicine, and is re-proposed by Wilhelm and colleagues in their work. But this feature does not specifically characterize the homeopathic methodology.
